# Vaccine Escape Recombinants Emerge after Pneumococcal Vaccination in the United States

**DOI:** 10.1371/journal.ppat.0030168

**Published:** 2007-11-16

**Authors:** Angela B Brueggemann, Rekha Pai, Derrick W Crook, Bernard Beall

**Affiliations:** 1 Department of Zoology, University of Oxford, Oxford, United Kingdom; 2 Department of Gastrointestinal Sciences, Christian Medical College, Vellore, India; 3 Nuffield Department of Clinical Laboratory Sciences, University of Oxford, Oxford, United Kingdom; 4 Centers for Disease Control, Atlanta, Georgia, United States of America; Children's Hospital Boston, United States of America

## Abstract

The heptavalent pneumococcal conjugate vaccine (PCV7) was introduced in the United States (US) in 2000 and has significantly reduced invasive pneumococcal disease; however, the incidence of nonvaccine serotype invasive disease, particularly due to serotype 19A, has increased. The serotype 19A increase can be explained in part by expansion of a genotype that has been circulating in the US prior to vaccine implementation (and other countries since at least 1990), but also by the emergence of a novel “vaccine escape recombinant” pneumococcal strain. This strain has a genotype that previously was only associated with vaccine serotype 4, but now expresses a nonvaccine serotype 19A capsule. Based on prior evidence for capsular switching by recombination at the capsular locus, the genetic event that resulted in this novel serotype/genotype combination might be identifiable from the DNA sequence of individual pneumococcal strains. Therefore, the aim of this study was to characterise the putative recombinational event(s) at the capsular locus that resulted in the change from a vaccine to a nonvaccine capsular type. Sequencing the capsular locus flanking regions of 51 vaccine escape (progeny), recipient, and putative donor pneumococci revealed a 39 kb recombinational fragment, which included the capsular locus, flanking regions, and two adjacent penicillin-binding proteins, and thus resulted in a capsular switch and penicillin nonsusceptibility in a single genetic event. Since 2003, 37 such vaccine escape strains have been detected, some of which had evolved further. Furthermore, two new types of serotype 19A vaccine escape strains emerged in 2005. To our knowledge, this is the first time a single recombinational event has been documented in vivo that resulted in both a change of serotype and penicillin nonsusceptibility. Vaccine escape by genetic recombination at the capsular locus has the potential to reduce PCV7 effectiveness in the longer term.

## Author Summary

The 7-valent pneumococcal conjugate vaccine is a remarkable public health success story. It has significantly reduced invasive pneumococcal disease in the United States not only by protecting vaccinated children, but also by protecting unvaccinated older children and adults by herd immunity. However, there was always a concern that use of a limited-valency vaccine would result in an increase in disease due to nonvaccine serotypes, and this has now occurred in the US. The predominant nonvaccine serotype causing invasive disease is 19A, and this increase is partially explained by “vaccine escape” pneumococci, strains that have exchanged the genes that encode a vaccine serotype 4 capsule for genes that encode a nonvaccine serotype 19A capsule. These strains are then able to escape vaccine-induced immunity. Characterisation of the genetic event that resulted in these vaccine escape strains was the focus of our study and the results were surprising. The results of this study have important relevance to the long-term effectiveness of the current vaccine and to the development of future pneumococcal vaccines.

## Introduction

Streptococcus pneumoniae (the “pneumococcus”) is one of the most important bacterial pathogens worldwide, especially among children. Pneumococcal pneumonia, meningitis, and septicemia result in 1 million deaths annually among children <5 y of age [1]. PCV7 protects against seven pneumococcal capsular types (serotypes)—4, 6B, 9V, 14, 18C, 19F, and 23F [2]—and has been used to vaccinate children in the United States since 2000. PCV7 has been remarkably effective in reducing disease among vaccinated children, and even among unvaccinated children and adults as a result of a striking herd immunity effect, due to the disruption of pneumococcal transmission from young children to older children and adults [2–6]. PCV7 is increasingly being used to vaccinate children in other countries.

There are 91 known pneumococcal serotypes, the last of which was discovered recently [7,8]. The ecological niche for the vast majority of the pneumococcal population is the nasopharynx of healthy children [9]; thus, any serotype-specific vaccine that is of limited valency and affects nasopharyngeal carriage will perturb the composition of the circulating pneumococcal population, with unknown consequences. The capsule is the principal known virulence factor with respect to invasive pneumococcal disease [10], and population biology studies indicated that certain serotypes have a greater potential to cause invasive disease than others [11,12]. Another important aspect of pneumococcal biology is that there is a strong association between genotype as defined by multilocus sequence typing (MLST), and serotype—that is, strains with the same MLST sequence type (ST) are usually of the same serotype [11,13,14].

Since the capsule is the principal invasive disease determinant, and is the target for serotype-specific prevention of disease by vaccination, there are two events that are especially important with respect to understanding vaccine effects: serotype replacement and capsular switching. At a population level, serotype replacement simply refers to a decrease in the prevalence of vaccine serotype pneumococci in the nasopharynx accompanied by a corresponding increase in nonvaccine serotype pneumococci, as they fill the newly vacant ecological niche. Serotype replacement in the nasopharynx of a healthy child may or may not be problematic; the public health concern is whether or not replacement serotypes also cause *disease*. Serotype replacement invasive disease among children and adults has significantly increased in the US post-vaccination [15–18], and invasive pneumococcal disease among children <5 y of age is now predominantly due to serotype 19A [17]. A recent *JAMA* report described a significant overall increase in nonvaccine serotype disease among Native Alaskan children, most frequently due to serotype 19A [18]. Genotypic characterisation of these serotype 19A strains by MLST showed that most of the serotype 19A replacement disease can be explained by clonal expansion of one genotype, ST199, which existed prior to vaccination [15,18].

The second major vaccine-related concern is the possibility of capsular switching, when the genes encoding one type of capsule are exchanged, via transformation and recombination, for the genes encoding a different type of capsule. Capsular switches from one vaccine serotype to another were first described 16 y ago [19–21], but it is the vaccine-to-*nonvaccine* serotype switch that is of primary concern, because it contributes to serotype replacement and allows for the possibility of vaccine escape. Acquisition of a nonvaccine capsule by a pneumococcal strain capable of causing invasive disease has been a serious concern related to the use of any serotype-specific vaccine [11,22].

**Figure 1:**
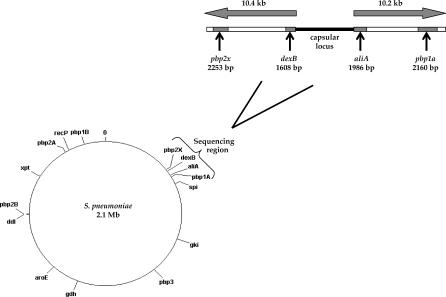
Schematic of the Pneumococcal Genome Housekeeping genes characterised in the MLST scheme (*aroE*, *gdh*, *gki*, *recP*, *spi*, *xpt*, and *ddl*), the genes for PBPs (*pbp2x*, -*1a*, -*3*, -*2b*, -*2a*, and -*1b*), the capsular locus flanked by the *dexB* and *aliA* genes, and the region around the capsular locus that was sequenced in this study (shown also in expanded version) are depicted. The capsular loci of serotypes 4 and 19A are 20.9 kb and 18.6 kb, respectively, in length.

The capsular locus of the pneumococcus is located between two genes, *dexB* and *aliA* ([7]; Figure 1). (Serotype 37 is one exception, as it has a defective capsular locus and the serotype is determined by the type 37 synthase gene [*tts*] located elsewhere in the pneumococcal genome [7,23].) Two of the six penicillin-binding proteins (PBPs) possessed by the pneumococcus are near the capsular locus: *pbp2x* is upstream of *dexB* and *pbp1a* is downstream of *aliA*. Alterations in PBPs confer penicillin resistance, and alterations in *pbp2x*, *pbp1a*, and *pbp2b* are the most important [24–26]. Penicillin-resistant pneumococci are a major problem throughout the world [27]; prior to use of PCV7 in the US, nearly one-quarter of invasive pneumococci were penicillin-nonsusceptible [28]. Initially, use of PCV7 in the US reduced the incidence of invasive disease due to antimicrobial-resistant vaccine serotypes, but more recently, antimicrobial resistance has increased with the increase of serotype 19A disease [28,29].

The Centers for Disease Control and Prevention (CDC) has been monitoring invasive pneumococcal disease since 1995 through the Active Bacterial Core (ABC) surveillance program [6,14,30] and as a result, the post-vaccination increase in nonvaccine serotype 19A disease in the US was quickly detected. Serotype 19A strains collected by the CDC through 2005 were genotyped by MLST, which revealed that vaccine escape strains had begun to emerge in 2003 [14,15]. These strains possessed an MLST genotype, ST695, that had always been associated with vaccine serotype 4 (ST695^4^), but now expressed a serotype 19A capsule (ST695^19A^). These strains were detected only 3 y after vaccine implementation, but rapidly increased in prevalence. The first three strains were detected in 2003; two strains were detected in 2004; and 32 strains were detected in 2005, some of which had evolved further. Moreover, in 2005, two new types of serotype 19A vaccine escape strains emerged, ST2365^19A^ (*n =* 4) and ST899^19A^ (*n =* 1); these appeared to represent new recombinational events that also occurred between serotype 4 recipients and serotype 19A donors. The aim of this study was to sequence the regions upstream and downstream of the capsular locus, including both PBPs, to identify the putative recombinational event(s) that resulted in these vaccine escape strains.

## Results

### Recipient, Donor, and Progeny Pneumococcal Strains Selected for Characterisation

Strains were selected based upon serotyping and MLST genotyping data and are described in Table 1. Between 1998 and 2005, 31,669 cases of invasive pneumococcal disease among all ages were identified from ABC sites, from which 28,363 pneumococcal strains were available for serotyping. 15,736 (55%) strains were of nonvaccine serotypes, and 1,935 of those were serotype 19A, 88% of which were recovered from 2000 to 2005 ([17] and ABC surveillance program, unpublished data). The following serotype 19A strains were genotyped by MLST: 82 of 113 (73%) strains from 1999 [14,31] and 779 of 1,597 (49%) strains from 2001 to 2005 ([14,15] and ABC surveillance program, unpublished data). No serotype 19A strains from 1998 or 2000 were genotyped by MLST. Possible serotype 19A capsular switches were identified among 57 of 779 (7%) of 2001–2005 genotyped strains: ST271, 690, 899, 1092, and 1790 (*n* = 1 strain each); ST236, 338, and 557 (*n* = 2 strains each); ST230 and 292 (*n* = 3 strains each); ST156 (*n* = 4 strains); and ST695 (*n* = 37 strains) [14,15,31]. Among these strains, the ST695^19A^ plus related ST899^19A^ strains were characterised in this study. All other possible capsular switch strains were present in very low numbers and/or were also found outside the US; vaccine and nonvaccine serotype variants of ST156 are recognised in the global database (http://www.mlst.net/).

**Table 1:**
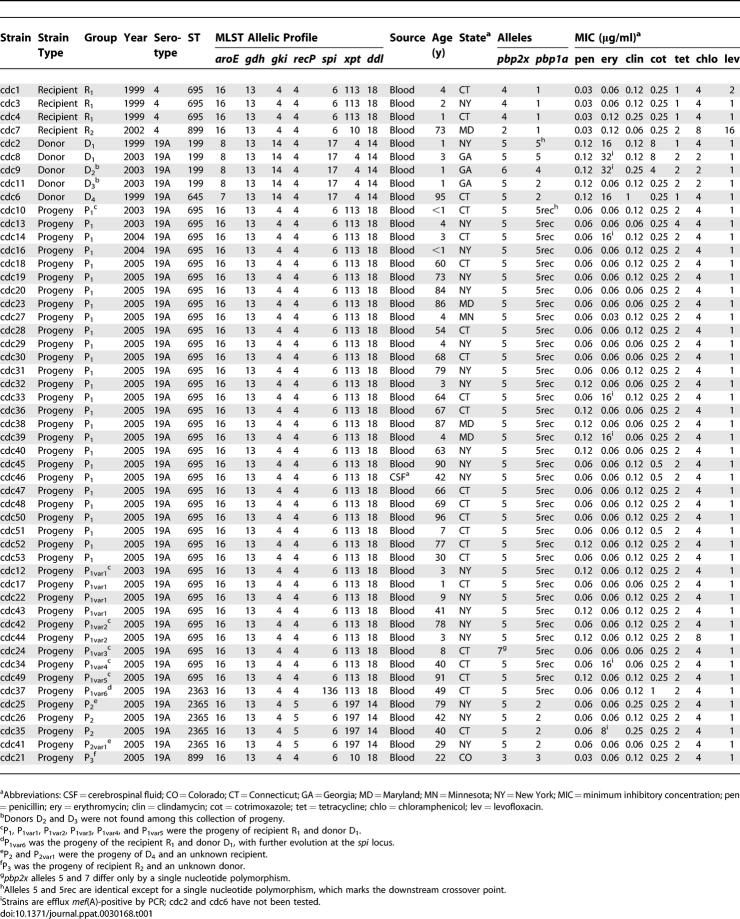
Description of the Pneumococcal Strains Characterised in This Study

Recipient strains were those with the background genotype into which the 19A capsular locus recombined: ST695^4^ (*n =* 3), recovered in 1999, and ST899^4^ (*n =* 1), recovered in 2002. Based upon pulsed-field gel electrophoresis, selected MLST data, and eBURST analysis, it is likely that strains of ST899^4^ were also common among children <5 y of age before vaccine introduction [14]. Putative donor strains of the 19A capsular locus were selected from pneumococci recovered in 1999 and 2003. The most likely donor of the 19A capsular locus was ST199^19A^, because ST199^19A^ existed as a prevalent strain pre-vaccine implementation and was the major clone responsible for serotype replacement disease post-vaccine implementation [15,17]. Thus, four pre- and post-vaccine implementation strains of ST199^19A^ were selected, plus one strain of ST645^19A^, which is a single-locus MLST variant of ST199^19A^. All serotype 19A strains collected through 2005 that were ST695 or closely related MLST genotypes were characterised as progeny strains. This resulted in the characterisation of 42 unique strains: ST695^19A^ (*n =* 36), ST2365^19A^ (*n =* 4), ST2363^19A^ (*n =* 1), and ST899^19A^ (*n =* 1).

Pneumococci were recovered from patients aged <1 to 96 y with invasive pneumococcal disease (Table 1). All pneumococci were recovered from blood, apart from one strain (cdc46) that was recovered from cerebrospinal fluid. Among vaccine escape strains, 40 of 42 (95%) were collected in the northeastern US (note that 37% of all 20,196 available isolates since 2000 were from the Northeast; ABC surveillance program, unpublished data); and two strains were from Minnesota (cdc27) and Colorado (cdc21). Three putative donor strains were from Georgia (cdc8, cdc9, and cdc11); all other putative donor and recipient strains were collected in the Northeast. All vaccine escape strains were recovered from 2003 to 2005. Strains from 2003 to 2004 were recovered from children ≤4 y of age, although only pediatric isolates were genotyped [15]; 2005 strains were recovered from children and adults.

### Sequence Homology within Regions Flanking the Capsular Locus

Regions upstream and downstream of the capsular locus were sequenced in all 51 pneumococci (Figure 1). Sequence alignment comparisons showed that recipient, donor, and progeny strains could be grouped based upon the relatedness of the regions flanking the capsular locus, considered as one joined sequence of 20.6 kb (Figure 1). The capsular locus has not yet been sequenced, but serotypes have been confirmed phenotypically, and the serotypes of a subset of the vaccine escape strains were confirmed by a PCR assay [32]. The capsular locus is considered here only as it encodes either a serotype 4 or 19A capsule.

Progeny strains consisted of three groups, P_1_, P_2_, and P_3_ (Table 1). Twenty-seven ST695^19A^ progeny strains (P_1_) were identical to each other in the capsular locus flanking regions and were the major group of vaccine escape strains. There were five additional P_1_ variants, all of which differed from the P_1_ strains by only one to two single nucleotide polymorphisms (SNPs) over the entire region of sequence. Furthermore, one strain, P_1var6_, was a single-locus MLST variant of the P_1_ strains at *spi*, but the capsular locus flanking regions of P_1_ and P_1var6_ were identical. P_2_ progeny strains and strain P_3_ were 0.3% and 1%, respectively, divergent from P_1_ progeny over the entire region of sequence.

Among recipient strains, the flanking sequences of cdc1, cdc3, and cdc4 (R_1_) were identical; recipient strain cdc7 (R_2_) was 0.5% divergent from R_1_ strains (Table 1). Among 19A donor strains there were four unique sequences: cdc2 and cdc8 (D_1_), cdc9 (D_2_), cdc11 (D_3_), and cdc6 (D_4_).

### Recombinational Crossover Points and Likely Serotype 19A Donors among Progeny Strains

**Figure 2:**
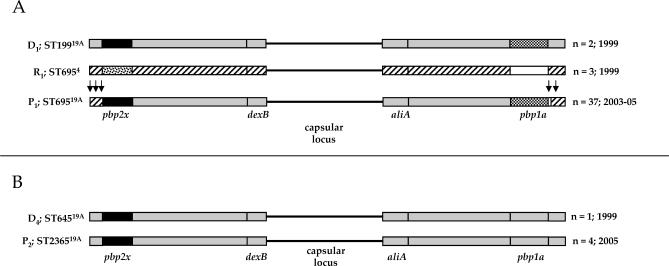
Sequencing Results among Progeny, Donor, and Recipient Pneumococci, including Differences in *pbp2x* and *pbp1a* Genes P_1_ and P_1variants_, and the P_2_ and P_2variant_, are each represented by one P_1_ and P_2_ schematic, respectively, for simplicity. Unique regions of sequence are indicated by variations of shading and patterns, e.g. D_1_, P_1_, D_4_, P_2_ all have the same *pbp2x* sequence (black), whereas R_1_ has a different *pbp2x* sequence (speckled). (A) Schematic of the recombinational crossover points revealed in the major group of progeny strains, ST695^19A^. Three SNPs marked and confirmed the upstream crossover region and two SNPs marked and confirmed the downstream crossover region in the progeny strains, as shown with black arrows. The length of the recombinational fragment was 38.6 kb (10.3 kb upstream + 18.6 kb capsular locus + 9.7 kb downstream). (B) Illustration of the second recombinational event. ST2365^19A^ is identical to ST645^19A^ in the entire sequenced region; no obvious crossover points have yet been revealed and the recipient is unknown.

Sequence alignments of the capsular locus flanking regions revealed the upstream and downstream recombinational crossover points in the ST695^19A^ strains. Three SNPs (heterologous to donor and homologous to recipient) marked and confirmed the upstream crossover point, just upstream of *pbp2x*; similarly, two SNPs marked and confirmed the downstream crossover point, at the 3′ end and just beyond the 3′ end of *pbp1a* (Figures 2A and S1), resulting in a recombinational fragment of 38.6 kb. To our knowledge, this is the first reported in vivo example of a recombinational event in pneumococcus in which the capsular locus and both adjacent PBPs recombined in what appeared to be a single event. This was shown to be experimentally possible by Trzcinski and colleagues [33].

The likely donor of the serotype 19A capsular locus and flanking regions in ST695^19A^ was ST199^19A^. Although the actual strain that donated the DNA cannot be identified with certainty, it is represented by the pre- and post-vaccination D_1_ strains from 1999 and 2003, respectively (Figures 2A and S1).

Sequence alignments also revealed a second recombinational event around the capsular locus in four strains of P_2_ progeny, all of which were first identified in 2005. Three P_2_ strains were identical (or one SNP different, P_2var1_) to D_4_ in the entire flanking region sequenced (Figures 2B and S1), suggesting that D_4_ was the likely donor of the 19A capsule among P_2_ progeny strains.

The third type of 19A progeny strain identified in this study was P_3_ (ST899^19A^). ST899 appears to have circulated at least since 1999 as a serotype 4 strain [14], but in 2005 appeared as a 19A serotype strain (Table 1). None of the sequenced 19A donors in this study share distinctive regions of homology to the P_3_ progeny; thus, the donor of this 19A capsular locus is still unknown.

### Relatedness Based on MLST Genotype

**Figure 3:**
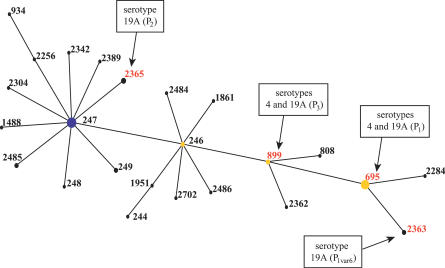
eBURST Analysis of Closely Related STs Found in This Study and in the MLST Database (http://www.mlst.net/) as of March 2007 STs described in the current study are indicated with arrows and boxes. The size of each circle is proportional to the number of isolates of that ST, the blue circle indicates the founder, ST247, of the entire clonal complex, and the yellow circles indicate subgroup founders (STs that have at least two single-locus variants). Strains from the ST247 founder complex have been recovered throughout Europe, South America, and the US (ST2365 recovered only in the US), from 1978 to 2005. The allelic profile of ST247 is *aroE* 16, *gdh* 13, *gki* 4, *recP* 5, *spi* 6, *xpt* 10, and *ddl* 14. Strains from the ST246 subgroup have been detected in Spain, Australia, and the United Kingdom from 1997 to 2005; strains from the ST899 subgroup have been recovered in the US and the Czech Republic from 1999 to 2002; and strains of the ST695 subgroup have been detected in the US and South Africa from 1999 to 2005. Pneumococcal strains of all STs in this diagram are serotype 4 except where indicated; however, the serotype 19A strains have only been reported in the US to date, and only since 2003. P_1_, P_1var6_, P_2_, and P_3_ refer to the type of progeny strains (see Table 1).

Figure 3 further clarifies the evolution of these vaccine escape strains by depicting the relatedness of the background MLST genotypes. The P_1_ progeny (including P_1_ variants) are all in the cluster with ST695 as the founder genotype. ST695 is a single-locus variant of ST899, differing only at *xpt* (allele 113) as a result of a 39-codon deletion in the middle of the *xpt* gene. This truncated *xpt* allele was first identified in 1999 among US strains [31] and was present in ST2363^19A^ and ST2284^4^. ST2363 and ST2284 differ from ST695 at *spi* and *recP*, respectively. The truncated *xpt* allele is identical to *xpt* allele 10 apart from the large deletion, thus allele 113 probably derived from the full-length allele 10 (Table 1; http://www.mlst.net/).

The P_2_ progeny (including the P_2variant_) appeared to evolve from the founder ST247^4^ clonal complex. The oldest known strains of ST247^4^ were identified in The Netherlands in 1978, and ST247^4^ and the related single-locus variants in the ST247^4^ cluster have been detected in other European and South American countries through at least 2005 (Figure 3; http://www.mlst.net/). The P_2_ progeny were first identified in 2005 in the US. ST247^4^ strains have not yet been characterised, thus recombinational crossover points in P_2_ progeny have not yet been identified. The third serotype 19A capsular change was the P_3_ progeny (ST899^19A^); Figure 3 depicts the relatedness of this ST to other STs in the clonal complex.

### Sequence Diversity among PBPs

**Figure 4:**
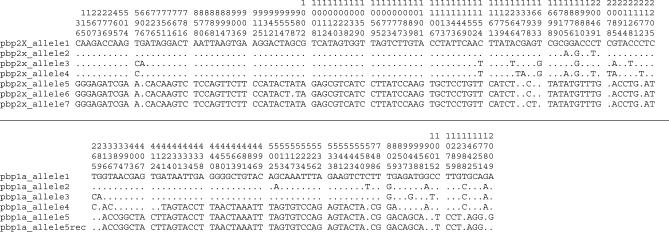
Sequence Alignments of *pbp2x* and *pbp1a* (2,253 and 2,160 Base Pairs, Respectively, in Total Length), Showing the Polymorphic Sites (Numbered Vertically) in Each of the PBP Alleles Present in This Dataset Note that *pbp1a* is reverse complemented. Allele 1 for each gene corresponds to the penicillin-susceptible TIGR4 pneumococcal strain [38]. *Pbp1a*_allele 5 and *pbp1a*_allele 5rec are identical apart from base pairs 1784 and 2009, which mark the downstream recombinational crossover point in progeny strains ST695^19A^.

Alterations in PBP genes *pbp2x*, *pbp1a*, and *pbp2b* confer penicillin resistance [24–26]. Allele assignments were given to each unique *pbp2x* and *pbp1a* sequence among this collection of strains; the *pbp2x* and *pbp1a* sequences from TIGR4, a serotype 4 penicillin-susceptible strain, were used for comparison and designated allele 1 (Table 1; Figure 4). All R_1_, R_2_, and P_3_ strains possessed *pbp2x* and *pbp1a* alleles similar to those in TIGR4, which correspond to the basal penicillin minimum inhibitory concentration (MIC) of 0.03 μg/ml that is typical of all ST695^4^ isolates. Among D_3_, D_4_, and P_2_ strains, *pbp2x* was altered while *pbp1a* was largely conserved; both *pbp2x* and *pbp1a* were altered in the D_1_, D_2_, P_1_, and P_1variant_ strains. Phenotypically, strains with altered *pbp2x* and *pbp1a* genes resulted in a MIC to penicillin of 0.06–0.12 μg/ml (Table 1). The breakpoints for penicillin resistance are ≤0.06 μg/ml (susceptible), 0.12–1.0 μg/ml (intermediate resistance), and ≥2 μg/ml (high-level resistance), but the variation in susceptibility testing is ±1 doubling dilution [34]. All donor strains and all progeny strains apart from the P_3_ strain had MIC values at the penicillin-susceptible breakpoint; however, based on the *pbp2x* and *pbp1a* sequences, D_1_, D_2_, P_1_, and P_1variants_ had divergent PBPs typical of penicillin-nonsusceptible strains ([24–26]; Figure 4). All *pbp* sequences in this study were unique to the National Center for Biotechnology Information database.

### Resistance to Other Antimicrobials

Among all strains, four donor and five vaccine escape strains were erythromycin resistant, possessing the *mef*(A) efflux phenotype [29,35]; all five vaccine escape strains and two donors (cdc8 and cdc9) were confirmed to be *mef*(A)-positive by PCR ([36,37]; Lesley McGee, Emory University, personal communication). The other donor strains were not tested by PCR. One erythromycin-resistant donor strain (D_4_) was also clindamycin resistant (Table 1). D_1_ and D_2_ donor strains were co-trimoxazole resistant, and one vaccine escape strain (cdc13) was resistant to tetracycline. One P_1var2_ strain (cdc44) was resistant to chloramphenicol; R_2_ was resistant to chloramphenicol and levofloxacin.

## Discussion

To our knowledge, this is the first report of vaccine escape events in the US subsequent to national pneumococcal vaccination. The main event resulted in nonvaccine serotype, penicillin-nonsusceptible ST695^19A^ pneumococci as a result of recombination between the recipient ST695^4^ and donor ST199^19A^ (D_1_). A second event resulted in the emergence of ST2365^19A^ from the well-established serotype 4 clonal complex of ST247, and these strains had a different serotype 19A donor, ST645^19A^. No recipient has yet been identified, but recipient strains of ST247 will be characterised to elucidate the recombinational crossover points in these progeny. Finally, a third event resulted in the emergence of ST899^19A^, but the serotype 19A donor is not yet known.

The most plausible explanation for the emergence of these strains is that one main recombinational event, ST695^19A^, occurred around 2003, and the fact that it was a nonvaccine serotype variant provided the selective advantage by which these strains could evade the immune pressures invoked by the vaccine. Furthermore, with the additional selective advantage of penicillin nonsusceptibility, this progeny strain increased in frequency over the next 2 y, disseminating through the northeastern US to become the fourth most common serotype 19A genetic complex in the US (ABC surveillance program, unpublished data). The recombinational event did not appear to result in a decrease in fitness among these strains, as strains increased over time within the population. More worryingly, all of these strains were recovered from patients with bacteremia and meningitis. We cannot be absolutely certain that ST695^19A^ strains never existed pre-vaccination, but extensive surveillance pre- and post-vaccination in the US failed to reveal any such strains [14,31], and no such strains have been reported to the MLST database from other parts of the world. Hence, even if these strains did exist pre-vaccination they were likely to be very rare, and it could still be maintained that the immune pressure resulting from PCV7 use selected for the emergence of such strains.

The two additional progeny strain types, ST2365^19A^ and ST899^19A^, were identified in 2005, and this may be the very early detection of these new recombinants. Whether or not these strains will increase in prevalence remains to be seen. Since serotype 19A was the major replacement disease serotype to emerge post-vaccination [15,17], our efforts to understand the genetics of this event have been focused on serotype 19A strains. This will be expanded to include other putative capsular switching events to better understand the phenomenon of capsular switching. Although it has been known for many years that capsular switching can and does occur, it is not at all clear how often it occurs within the pneumococcal population.

An exciting component of the current study is that recombination in the pneumococcus has been revealed almost as it occurred. This presents a unique opportunity to measure recombination *in nature* as a result of vaccine-induced changes, and may shed light on how much recombination occurs in general within the pneumococcal genome. Studies to explore these strains in detail are ongoing.

What are the implications for vaccine escape? Clearly, vaccine escape by recombination at the capsular locus has the very real potential to reduce PCV7 effectiveness in the longer term. This will almost certainly be true for any serotype-specific pneumococcal vaccine, given the diversity and complexity of serotypes possessed by the pneumococcus. These US data reinforce two key points: i) the importance of surveillance pre- and post-vaccination in countries preparing to implement PCV7, to detect changes as and when they occur; and ii) the importance of understanding the genetic events that result in vaccine escape. Discerning the genetic event is crucial to understanding vaccine escape and pneumococcal recombination in general. Such knowledge will provide guidance about the design and use of future pneumococcal vaccines.

## Materials and Methods

Invasive pneumococcal strains were collected as part of the ABC surveillance program at the CDC. Microbiological testing and molecular characterisation by MLST was performed as previously described [14,31]. Strains for this study were selected from pre- and post-vaccine implementation ABC collections and sent to the University of Oxford. Sequence alignments of the TIGR4 [38], R6 [39], and Spain^23F-1^ (http://www.sanger.ac.uk/Projects/S_pneumoniae/) pneumococcal genomes were used to design 51 sets of PCR primers (available upon request) in regions upstream and downstream of the capsular locus (Figure 1).

DNA was extracted from pneumococcal strains using the DNeasy Tissue Kit (Qiagen UK). PCR assays were identical for all combinations of PCR primers: 2.5 μl of PCR buffer (Qiagen), 0.5 μl of dNTPs (200 μM stock), 0.5 μl of forward primer (100 μM stock), 0.5 μl of reverse primer (100 μM stock), 0.3 μl of Taq polymerase (Qiagen UK), 19.7 μl of nuclease-free water (Invitrogen UK), and 1 μl (∼1 μg) of extracted DNA. Thirty-five cycles of PCR amplification were performed: denaturation at 94 °C for 30 s, annealing at 55 °C for 30 s, and elongation at 72 °C for 60 s. Ten-microliter sequencing reactions were prepared with 4 μl primer (1 μM stock of PCR primers), 1.75 μl sequencing buffer (Applied Biosystems), 0.5 μl Big Dye (Applied Biosystems), 1.75 μl nuclease-free water, and 2 μl cleaned PCR amplicons; followed by 25 sequencing cycles of 96 °C for 10 s, 50 °C for 5 s, and 60 °C for 2 min. PCR amplicons and sequencing products were cleaned as described previously [15].

Sequences were assembled and edited using Pregap4 and Gap4 (Staden Package, http://staden.sourceforge.net/). Consensus sequences were aligned using Multalin [40] and trimmed to the longest possible consensus sequence shared by all strains. Trimmed sequences were uploaded into MEGA version 3.1 [41], and compared in an iterative process to ascertain which strains were donors of the 19A capsule and reveal recombinational crossover points in progeny strains. The eBURST algorithm was used to compare all progeny strains to all pneumococcal strains represented in the MLST database [42].

## Supporting Information

Figure S1 Sequence Alignments of Major Donor, Recipient, and Progeny Strains in This Study, Aligned with the Corresponding Region of the TIGR4 Sequence Polymorphic sites are shown across the entire sequenced region upstream and downstream of the capsular locus. Black arrows highlight the SNPs in the upstream and downstream crossover regions; the locations of *pbp2x*, *pbp1a*, and the (missing) capsular locus are also shown. Strains are labeled A and B to correspond to the strains involved in the two main recombinational events shown in Figure 2.
(95 KB PPT)

### Accession Numbers

All unique *pbp2x* and *pbp1a* sequences in this study were submitted to GenBank (http://www.ncbi.nlm.nih.gov/Genbank/index.html) under accession numbers EU034013–EU034023.

## Abbreviations

ABC: Active Bacterial Core
CDC: Centers for Disease Control and Prevention
MIC: minimum inhibitory concentration
MLST: multilocus sequence typing
PBP: penicillin-binding protein
SNP: single nucleotide polymorphism
ST: sequence type
